# Comparative Study of the Effects of Drugs Targeting Adrenergic Receptors on the Early Life Stages of Zebrafish

**DOI:** 10.3390/toxics12080583

**Published:** 2024-08-10

**Authors:** Junsheng Lv, Fengzhu Sun, Zaitian Li, Yueyun Qin, Ruozhu Sheng, Liwei Sun

**Affiliations:** College of Environment, Zhejiang University of Technology, 18 Chaowang Road, Hangzhou 310032, China

**Keywords:** adrenergic system, neurobehavior toxicity, cardiovascular response, biomarker, pharmaceuticals

## Abstract

Owing to the presence of drugs targeting adrenergic receptors in aquatic ecosystems, considerable attention has been directed towards their environmental distribution and fate in recent decades. However, their potential impacts on non-target aquatic organisms, particularly fish, have received relatively limited investigation. In this study, moxisylyte (MOX) and propranolol (PRO) were selected as representatives of α- or β-adrenergic receptor antagonist, respectively, and we assessed their effects on the early life stages of zebrafish, especially on the nervous and cardiovascular systems. Although both compounds exhibited marginal effects on zebrafish survival, hatching and gross abnormality following exposure to concentrations ranging from 1 to 625 μg/L, they adversely affected the development of cardiovascular and nervous systems, but through different mechanisms of action, as evidenced by variations in gene transcriptional responses and enzyme activities. Notably, cardiovascular responses appear promising for use as potential biomarkers for exposure to drugs targeting adrenergic receptors. This study enhances our understanding of the ecotoxicological risks posed by α- and β-blockers in fish. Nonetheless, further investigation is needed to elucidate the precise mechanisms underlying the impacts of drugs targeting adrenergic receptors due to our limited knowledge of the physiological functions of the adrenergic system in fish.

## 1. Introduction

Adrenergic receptors (ARs), also known as adrenoceptors, are members of the G protein-coupled receptors (GPCRs) superfamily, responsible for mediating physiological responses to catecholamines, such as norepinephrine (noradrenaline) and epinephrine (adrenaline) [1]. The adrenergic system plays a crucial role in regulating endocrine, neuronal, autonomic, cardiovascular, and metabolic functions, underscoring the significance of adrenergic receptors in maintaining homeostasis [1,2].

Based on their relative affinity for characteristic ligands, adrenergic receptors are classified into two main types, α- and β-adrenergic receptors. Each type can be further divided into subtypes according to their pharmacological and physiological properties [1]. Adrenergic receptors are widely expressed in various tissues and organs throughout the body, making them promising therapeutic targets [1,3]. A wide range of drugs targeting adrenergic receptors, including agonists and antagonists, have been developed and are extensively used due to their efficacy in treating a broad spectrum of cardiovascular, respiratory, and metabolic disorders [3,4]. β-adrenergic receptor antagonists, or β-blockers, for instance, have been developed and extensively used for managing various cardiovascular conditions, including arrhythmias, hypertension, angina pectoris, and heart failure [5,6]. Over thirty types of β-blockers have been used clinically, with several ranking among the most prescribed and consumed medications worldwide [5,7]. Furthermore, the high incidence of cardiovascular disease is expected to fuel the growth of the β-blockers market in the forecast period.

Since the initial evidence reported in the 1970–1990s, the presence of pharmaceuticals in the aquatic environment has become a heightened concern in recent decades [8]. Pharmaceuticals are designed for their biological activities, making their potential impacts on non-target aquatic organisms more significant compared to certain conventional pollutants. These pharmaceuticals and/or their metabolites are continuously discharged into the environment, particularly the aquatic environment, in large amounts from drug manufacturing, patient and animal excretion, aquafarming, and the disposal of unused or expired medicines, rendering many of them “pseudo-persistent” [8,9]. Due to their widespread use and limited metabolism in humans, drugs targeting adrenergic receptors have been detected in various aquatic environments, including wastewater treatment plant (WWTP) effluents and influents, surface water, groundwater, and even drinking water [5,10,11]. For example, β-blockers are among the most frequently detected therapeutic classes, and at least twelve β-blockers have been identified in various aquatic environments, with concentrations ranging from nanograms per liter to several hundred micrograms per liter [11,12]. The highest concentration reported was up to 307 μg/L for atenolol in wastewater influent in India, as reviewed by Yi et al. [11].

The adrenergic system is conserved across vertebrate families. Recent but limited data have revealed that fish and mammalian androgenic receptors exhibit remarkable similarities, suggesting a wide range of functions for the adrenergic system and the catecholamines in fish [5,7]. Consequently, it is reasonable to infer that drugs targeting adrenergic receptors may induce similar pharmacological and toxicological effects in fish. Over the past few decades, extensive research, including our own, has examined the adverse impacts of drugs targeting adrenergic receptors on fish mortality, growth, reproduction, cardiac development, and the endocrine system [5,11,12,13,14,15], though results have been partially conflicting. Moreover, research efforts have primarily focused on β-blockers, given their dominance in the pharmaceutical market and frequent presence in the aquatic environment, while the impacts of other drugs targeting adrenergic receptors remain less understood.

Therefore, this study aimed to investigate the toxicological effects of drugs targeting adrenergic receptors in fish. Moxisylyte, a specific α-adrenergic antagonist, has been approved for medical use since 1987 [15]. Propranolol, a non-selective β-adrenergic antagonist, has been approved since 1964 [14]. They were selected as representatives of α-blockers and β-blockers, respectively. Their effects on the early life stages of zebrafish, including conventional toxicity endpoints such as mortality and time to hatching, were assessed and compared. Furthermore, their impacts on the nervous and cardiovascular system were evaluated, considering the current, albeit limited, knowledge of the physiological function of the adrenergic system in fish. The results are expected to enhance our understanding of the effects of different drugs targeting adrenergic receptors on aquatic organisms, thus contributing to the ecotoxicological risk assessment of these pharmaceuticals.

## 2. Materials and Methods

### 2.1. Chemicals

Moxisylyte hydrochloride (MOX, CAS: 964-52-3) and propranolol hydrochloride (PRO, CAS: 318-98-9) with purities exceeding 99% were purchased from Sigma-Aldrich, St. Louis, MO, USA. Stock solutions were prepared without the need for carrier solvent due to the high solubility of these test substances.

### 2.2. Zebrafish Maintenance and Embryo Production

Wide-type AB zebrafish (*Danio rerio*) were sourced from the China Zebrafish Resource Center (Wuhan, China), and maintained following established protocols [16]. Embryo production was conducted following the methods described by Westerfield [17]. Briefly, adult males and females were collocated in the breeding tanks one hour before the onset of light. Subsequently, embryos were collected, washed, and visually inspected under an inverted microscope, and those exhibiting normal development were chosen for the following testing procedures.

### 2.3. Exposure Conditions for Embryos and Larvae

At 2 h post-fertilization (hpf), embryos were transferred to glass beakers containing 200 mL of test solutions. Each treatment comprised three replicates, totaling 240 embryos per treatment. The exposure concentrations for MOX and PRO were 1, 5, 25, 125 and 625 μg/L. Control groups, exposed solely to dechlorinated tap water, were included. Fish were maintained at a constant temperature of 28 ± 1 °C with a photoperiod of 16 h light to 8 h dark. Exposure lasted 5 days, with daily solution renewals. Observations were made at least three times daily. The dead fish, identified by embryo coagulation, loss of heartbeat, lack of somite formation, and non-detachment of the tail [18] were removed as soon as observed. The hatching period of zebrafish occurs between 48 and 72 hpf, and the hatching time was recorded when the larvae emerged. The fish morphology was also visually evaluated, and developmental malformations, such as yolk sac edema, pericardial edema, curved or bent body axis, collectively referred to as “gross abnormality” (Figure S1), were recorded. Dead or malformed fish were excluded from the subsequent tests on the cardiovascular and nervous systems.

The survival rate was defined as the percentage of surviving fish among the total exposed individuals from each replicate. Hatchability at 72 hpf was defined as the percentage of fish hatched before 72 hpf among the total hatched fish before the end of exposure (120 hpf). Time to hatching was defined as the sum of the time to hatching for each hatched fish divided by the total number of hatched fish. Gross abnormality rate was defined as the percentage of individuals with morphological malformations among the total number of exposed fish from each replicate.

The chemical analysis for PRO followed a previously described method [19]. Briefly, the exposure solutions were sampled and extracted using activated Sep-Pak C18 cartridges. PRO was then eluted with methanol and dried under nitrogen. Quantification was performed using a reverse-phase liquid chromatography system equipped with a C18 reverse-phase column. Separation employed an HPLC isocratic system elution with a mobile phase composed of 28:72 (*v*/*v*) acetonitrile:acidic water (1% acetic acid and 0.2% triethylamine). PRO samples were reconstituted in the mobile phase, and then injected. Peaks were monitored using a fluorescence detector with an excitation wavelength of 296 nm and an emission wavelength of 340 nm. As the test solutions were renewed daily, actual concentrations were measured before and after renewal. The average percent recovery was 96.8% for freshly prepared test solutions with different nominal concentrations, and 87.6% for the test solutions prior to renewal, which closely matched the nominal values. However, no analytical method is currently available for MOX due to its high biodegradability [20]. Although MOX concentrations were not measured, procedures were followed to minimize the effects of moxisylyte instability, as described previously [15]. For simplicity, nominal concentrations of PRO and MOX were used in this study.

### 2.4. The Measurement of Heartbeat, Blood Flow and Vessel Diameter

At 48 hpf, fifteen fish were randomly sampled from each replicate to measure cardiovascular parameters, including heartbeat, blood flow, and vessel diameter. For unhatched embryos, the chorion was removed with tweezers before measurement. The measurements were performed on the main artery using the ZebraLab VideoTrack system (Blood Flow V1.0.1, ViewPoint Life Science, Civrieux, France) according to the manufacturer’s protocol.

### 2.5. Locomotor Behavior Assay

After five days of exposure, fifteen larvae from each of three replicates were individually transferred into wells of 96-well plates containing 100 μL dechlorinated tap water, and then acclimatize for about 10 min. Locomotor behavior was assessed using the Zebralab VideoTrack system, as described previously [21,22]. The swimming responses of fish during light–dark transition (5 min light acclimation, 5 min dark stimulation, 5 min dark acclimation, and 5 min light acclimation) were recorded every 60 s. The swimming speed data were then analyzed and compared across each of the 5 min light/dark phases [22].

### 2.6. Gene Transcription Analysis

After exposure, about fifteen larvae from each of three replicates per treatment were homogenized for gene transcription analysis. Total RNA was extracted using RNAiso Plus (Takara, Dalian, China). The RNA concentration was determined at 260 nm using an ultramicro UV-Vis spectrophotometer (Bioteke, Beijing, China), and its quality was verified by measuring the 260/280 nm ratio and through agarose gel electrophoresis. The RNA was then converted to first-strand cDNA using the ReverTra Ace qPCR RT kit (Toyobo, Osaka, Japan), followed by quantitative real-time PCR with SYBR green detection (Toyobo, Osaka, Japan) on a Mastercycler ep Realplex system (Eppendorf, Hamburg, Germany). Samples were run in triplicate with the following cycling parameters: denaturation for 1 min at 95 °C, followed by 40 cycles of 15 s at 95 °C, and 1 min at 60 °C. The primer sequences for the target genes related to the nervous and cardiovascular system were identical to those reported previously [23,24,25]. The *β-actin* gene served as the internal reference for normalization, and relative transcription was quantified using the comparative cycle threshold (Ct) method [26].

### 2.7. Acetylcholinesterase (AChE) Activity Measurement

After the exposure period, about thirty larvae from each of three replicates were homogenized on ice in normal saline. After centrifugation at 4 °C, the supernatant was used to assess AChE activity using a chemical colorimetric method with a commercial kit, following the manufacturer’s instructions (Nanjing Jiancheng Bioengineering Institute, Nanjing, China). This assay relies on the production of a yellow color product (trinitrobenzene) resulting from the hydrolysis of acetylthiocholine by AChE. The optical density of this product was measured at 412 nm using a spectrophotometer (Multiskan Sky, Thermo Scientific, Waltham, MA, USA). Total protein concentration was determined using the bicinchoninic acid (BCA) method. Enzyme activity was expressed as nanomoles per minute per milligram of protein, and then normalized to the corresponding controls.

### 2.8. Data Analysis

The data were checked for normality and homogeneity of variance using the Kolmogorov–Smirnov test and Levene’s test, respectively. Subsequently, a one-way analysis of variance (ANOVA) followed by Dunnett’s post hoc test was performed using SPSS 19.0 software. The threshold for statistical significance was set at *p* < 0.05.

## 3. Results

### 3.1. Effects on the Embryonic Development of Zebrafish

No significant changes in mortality were observed for zebrafish embryos following exposure to MOX and PRO compared to the control group (Table S1). Exposure to MOX did not affect the hatchability or time to hatching, but increased gross abnormality relative to the controls, with statistical significance at 625 μg/L. Conversely, PRO did not affect hatchability or gross abnormality, but a significant decrease was observed in time to hatching at 5 μg/L.

### 3.2. Effects on the Locomotor Behavior of Zebrafish

**Figure 1:**
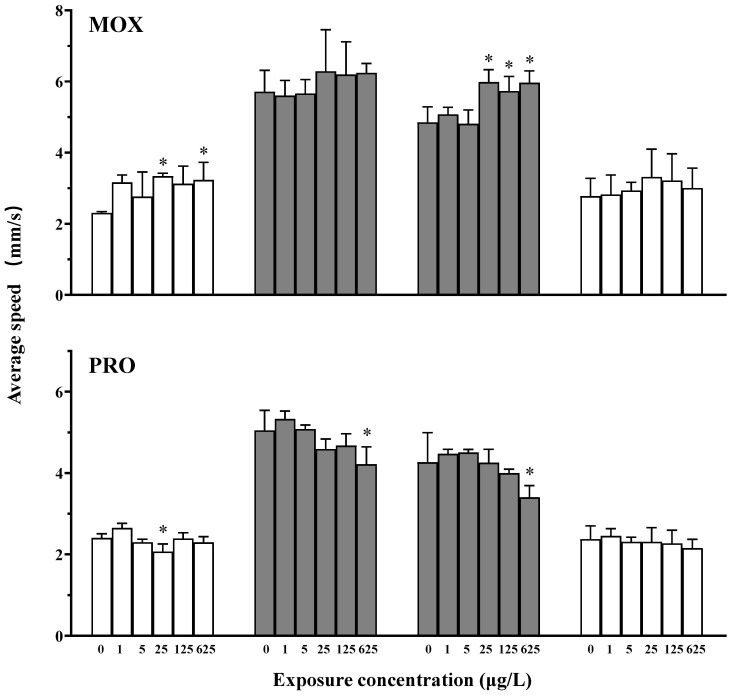
Changes in the swimming speeds of zebrafish larvae in light–dark photomotor response assays following exposure to moxisylyte (MOX) and propranolol (PRO). The white columns represent data from the light phase, while the black columns represent data from the dark phase. The values are expressed as the mean ± S.D. (three replicates with fifteen larvae each). Asterisks denote significant differences compared to the controls (* *p* < 0.05).

Exposure to MOX changed the swimming speed of larvae in response to a light–dark transition (Figure 1). Specifically, MOX exposure induced significant hyperactivity during the initial light acclimation phase at 25 and 625 μg/L, and during the dark acclimation phase at concentrations of 25 μg/L and above; however, no effects were observed in other phases.

Following exposure to PRO, a trend of decreasing swimming speed was observed (Figure 1). During the initial light acclimation phase, a significant hypoactivity was noted at 25 μg/L compared to the controls. Conversely, during both the dark stimulation and acclimation phases, locomotor activity decreased with increasing concentrations of PRO, with a significant difference observed at 625 μg/L. No effect was observed during the light acclimation phase.

### 3.3. Effects on the Heartbeat, Blood Flow and Vessel Diameter of Zebrafish

Exposure to MOX resulted in a reduction in vessel diameter and blood flow volume at a concentration of 625 μg/L compared to the controls (Table 1). Regarding heart rate, a slight increase was observed at low concentrations, but it was not statistically significant. However, as the concentration increased, the heart rate decreased, with a significant difference noted at the highest concentration (625 μg/L).

For PRO, a concentration-dependent decrease was observed for all three endpoints (Table 1). Significant reductions in vessel diameter and blood flow volume were noted at concentrations of 125 μg/L and above, while the decrease in heart rate was significant at concentrations of 25 μg/L and higher.

### 3.4. Effects on AChE Activity of Zebrafish

**Figure 2:**
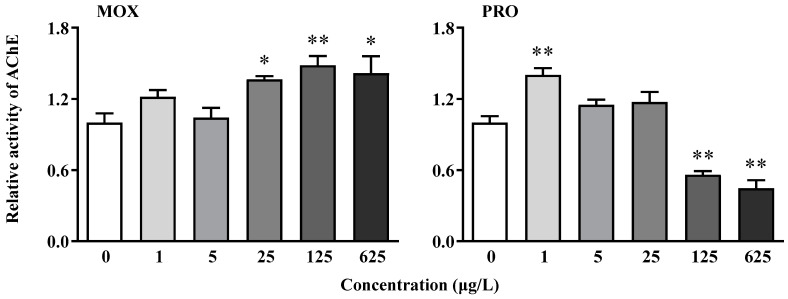
Changes in the activities of acetylcholinesterase (AChE) in zebrafish larvae following exposure to moxisylyte (MOX) and propranolol (PRO). The values relative to the controls are expressed as the mean ± S.D. (three replicates with thirty larvae each). Asterisks denote significant differences compared to the controls (* *p* < 0.05, ** *p* < 0.01).

Exposure to MOX led to increased AChE activity, with a significant difference observed at concentrations of 25 μg/L and above (Figure 2). Conversely, PRO exposure significantly induced activity at the lowest concentration (1 μg/L), followed by a decreasing trend with increasing concentration. Significant inhibition was observed at 125 and 625 μg/L (Figure 2).

### 3.5. Effects on Transcriptional Response of Genes Related to the Cardiovascular System in Zebrafish Larvae

**Figure 3:**
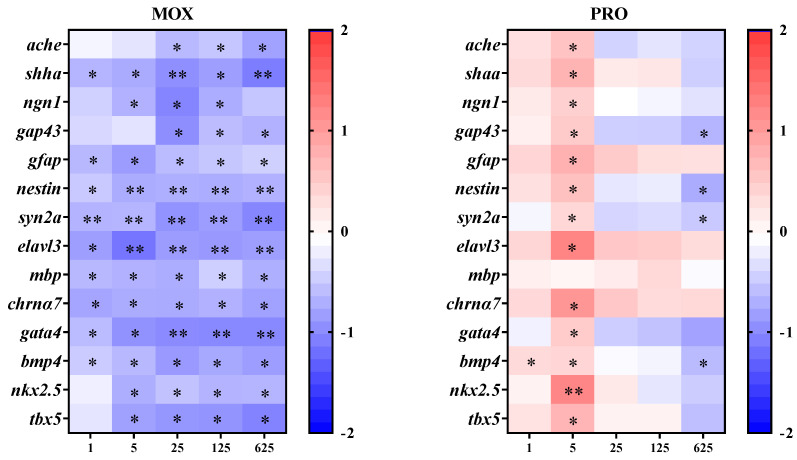
Transcriptional response of genes involved in the nervous and cardiovascular system in zebrafish larvae following exposure to moxisylyte (MOX) and propranolol (PRO). The average transcript values (three replicates with fifteen larvae each) relative to the controls are log2 transformed. Asterisks indicate significant differences compared to the controls: * *p* < 0.05 and ** *p* < 0.01.

MOX exposure significantly downregulated all four genes related to the cardiovascular system (Figure 3). Specifically, the transcription of *gata4* and *bmp4* showed significant decreases at all tested concentrations, while downregulation was observed at concentrations of 5 μg/L and above for *nkx2.5* and *tbx5*.

The transcription of *gata4* exhibited an inverted “U-shaped” response following exposure to PRO, with significant induction observed at 5 μg/L (Figure 3). Similar patterns were noted for the transcription of *nkx2.5* and *tbx5*. In the case of *bmp4*, PRO initially upregulated the transcription at 1 and 5 μg/L, followed by a concentration-dependent decrease, with significant downregulation evident at 625 μg/L (Figure 3).

### 3.6. Effects on Transcriptional Responses of Genes Related to the Nervous System in the Zebrafish Larvae

Following exposure to MOX, the transcription of *ache* decreased in a concentration-dependent manner, with significant differences observed at concentrations of 25 μg/L and above (Figure 3). Moreover, the transcription of genes related to neurodevelopment decreased following exposure to MOX, except for *ngn1* and *gap43* at certain concentrations (Figure 3).

Exposure to PRO elicited an inverted “U-shaped” response in the transcription of *ache*, with significant upregulation observed at 5 μg/L (Figure 3). Similarly, genes associated with neurodevelopment showed comparable inverted “U-shaped” trends, particularly for *shha*, *ngn1*, *gfap*, *elavl3*, and *chrnα7*. However, *gap43*, *nestin*, and *syn2a* exhibited significant transcriptional increases at 5 μg/L, followed by significant decreases at 625 μg/L. Conversely, PRO downregulated the transcription of *mbp* at the highest concentration (625 μg/L) (Figure 3).

## 4. Discussion

According to the ecotoxicity studies using phytoplankton, zooplankton, invertebrates, and fish, PRO exhibited the highest toxicity among all β-blockers [11]. Relatively few studies have focused on the toxicity of α-blockers, including MOX. In this study, both MOX and PRO had only marginal effects on the survival and development of zebrafish in the early life stages at the concentrations tested (Table S1). However, both compounds induced toxic effects on cardiovascular development in a concentration-dependent manner. MOX exposure at 625 μg/L significantly reduced vessel diameter, heart rate and blood flow volume, while PRO disrupted cardiovascular development at lower concentrations, reducing vessel diameter and blood flow volume at 125 μg/L, and reducing heart rate at 25 μg/L. These findings align with prior observations. Fraysse et al. [27] reported propranolol-induced reductions in zebrafish heart rate at 48 hpf, albeit at higher concentration (8 mg/L). Similarly, our previous study found that exposure to 1 mg/L of PRO significantly decreased zebrafish heart rate at 48 hpf, although effects at lower concentrations were not explored [13]. In a test based on medaka and zebrafish, Finn et al. [19] found no significant effects on heart rate in the embryos exposed to PRO at 0.1, 1, and 10 μg/L; however, the embryos from an exposed parent exhibited reduced heart rates across all developmental stages, accompanied by alterations in cardiac morphology, albeit with non-monotonic concentration–response curves. Larsson et al. [28] reported significant reductions in trout heart rate following the injection of 2 mg/kg PRO, while no effects on heart rate were found after 48 h of exposure to a very high PRO concentration (70 mg/L) in tank water. Comparable cardiovascular responses were observed in other aquatic organisms. For example, Dzialowski et al. [29] found that two β-blockers, PRO and metoprolol, concentration-dependently decreased heart rate in *D. magna*, an aquatic invertebrate. This observation was consistent with findings by Stanley et al. [30]. Therefore, despite uncertainty regarding effective concentration, the cardiovascular effects of β-blockers on development and function are unequivocal. Furthermore, regarding the β-blocker’s effects, cardiovascular development during embryonic stages was more sensitive than other endpoints, such as hatching rate, mortality or abnormality (Table S1), consistent with previous reports [7,13]. Based on knowledge of potential β-blocker mechanisms of action, Owen et al. [7] suggested that cardiovascular responses, such as heart rate or blood pressure, might be prioritized when identifying suitable exposure biomarkers for β-blockers. Regarding α-blockers, although targeting different androgenic receptor subtypes than β-blocker, they likely share common physiological functions, leading to similar pharmacological effects in some cases; for example, both are used to treat hypertension [1,7,15]. Therefore, it is not surprising that exposure to MOX, an α-blocker, could impact cardiovascular development and function as well.

The vertebrate heart’s development during embryogenesis is a complex process, characterized by a well-coordinated series of events and precise transcriptional regulation. The genes *gata4*, *tbx5*, *nkx2.5*, and *bmp4* encode key transcriptional regulators in cardiogenesis, and serve as biomarkers for assessing chemical cardiotoxicity [25]. The gene *gata4* encodes a zinc finger transcription factor, which plays a vital role in organogenesis, particularly in myocardial differentiation and function. The gene *nkx2.5* encodes a member of the NK homeobox gene family, which functions as a DNA-binding transcriptional activator implicated in multiple processes, including the regulation of cardiac chamber formation and circulatory system development. The gene *tbx5* encodes a T-box transcription factor involved in the formation of the cardiac conduction system and ventricular chambers during cardiogenesis. The gene *bmp4* encodes a protein from the bone morphogenetic protein family, which plays an important role in the left–right asymmetry of heart development [25]. In this study, exposure to PRO resulted in te significant upregulation of *gata4*, *nkx2.5*, and *tbx5* at 5 μg/L, and *bmp4* at 1 and 5 μg/L, followed by a concentration-dependent decrease, yielding an inverted “U-shaped” concentration–response relationship. Conversely, MOX exposure down-regulated the transcription of all four genes at nearly every concentration. The downregulation of key transcriptional regulators may explain the observed cardiovascular responses. Du et al. [25] reported that exposure to organophosphate flame retardants induced heart developmental toxicity, including arrhythmia and cardiac looping defects, during zebrafish embryogenesis, suggesting that *bmp4*, *nkx2.5*, and *tbx5* inhibition might contribute to heart developmental toxicity. However, due to the complexity of the transcriptional regulation network and the multifunctionality of certain genes like *bmp4* in various organogenesis processes, the further exploration of additional genes and their temporal–spatial transcription is necessary for a comprehensive understanding of the impact on cardiovascular development and function.

The adrenergic receptors are distributed throughout both the central and peripheral nervous systems [7]. Given the adrenergic system’s role in fish physiology, the modulation of the nervous system is directly influenced by the characteristics of α- or β-blockers. Furthermore, in comparison to mammals, the blood–brain barrier in fish is relatively permeable, facilitating the easier passage of xenobiotics [7]. Disruptions of nervous system development can have adverse effects on behavior and health, with locomotor behavior serving as a key indicator of nervous system development [21,22,24]. However, in this study, zebrafish larvae exhibited different locomotor responses to MOX and PRO exposure: MOX induced hyperactivity, while PRO induced hypoactivity in light–dark photomotor response assays. Previous reports have indicated that PRO can penetrate the blood–brain barrier in fish and decrease the neutral activity [7], thus the hypoactivity observed with PRO aligns with its expected mode of action. As for MOX, the hyperactivity may be attributed to the distinct physiological functions of α-adrenergic receptors, although the precise mechanism requires further investigation. Currently, there are limited data on the physiological functions of the adrenergic system in a small number of fish species, illustrating many similarities between fish and mammals, as well as differences [5,7]. This underscores the need for a deeper understanding of the physiology and pharmacology of adrenergic functions in fish.

Neurobehavioral responses encompass various physiological and biochemical processes. Disruption of the cholinergic system has been identified as a potential trigger for neurodevelopmental defects [21]. Acetylcholinesterase (AChE) is a pivotal enzyme responsible for catalyzing acetylcholine, a primary neurotransmitter in the cholinergic system, thereby terminating synaptic transmission. Consequently, enzyme activity and corresponding gene transcription are widely recognized as sensitive biomarkers for neurotoxicants [21,24]. PRO initially increased AChE activity at low concentrations, followed by a decrease with increasing concentration. Similarly, the transcription of the *ache* gene exhibited a similar trend, initially increasing before decreasing. However, the pattern of AChE activity and its transcription varied in response to MOX exposure. Despite inconsistencies between enzyme activity and gene transcription, it is suggested that both α and β-blockers impair acetylcholine-mediated neurotransmission in zebrafish larvae.

We further investigated the transcription of additional marker genes related to the nervous system to assess the neurotoxicity of the two compounds. The nicotinic acetylcholine receptor α-7, encoded by *chrna7*, plays a pivotal role in brain development, and regulates cognition, locomotion, and stress responses across the lifespan [24]. Five genes expressed in neuronal stem cells and/or developing neurons were selected: *nestin*, *elavl3*, *neurogenin1* (*ngn1*), *shha* (*sonic hedgehog a*) and *growth-associated protein 43* (*gap43*) [23]. Neuroepithelial stem cell protein (encoded by the *nestin* gene), an intermediate filament protein, is predominantly expressed in neuronal progenitor cells and neuronal stem cells during nervous system development, serving as a marker for stem cells committed to the neural fate [23,24]. The *elavl3* gene encodes the neuron-specific RNA-binding protein HuC, while *ngn1* encodes a helix-loop-helix transcription factor, both being implicated in regulating neurogenesis and identified as early neuronal markers in zebrafish and mammals [23,24,31]. Sonic hedgehog a (encoded by *shha*) acts as a morphogen that patterns many organ systems, including the nervous system [21,23]. Growth-associated protein 43 (encoded by *gap43*) is a nervous tissue-specific cytoplasmic protein, serving as an intrinsic determinant of neuronal development and plasticity [23]. Moreover, the synapsin IIa (encoded by *syn2a*) is a neuronal phosphoprotein crucial for neurotransmitter release and synaptogenesis, serving as a marker of synapse formation. Glial fibrillary acidic protein, encoded by the *gfap* gene, is an intermediate filament protein expressed in numerous cell types of the central nervous system, especially in astrocytes, and is regarded as a marker of astroglia [23]. Myelin basic protein (encoded by *mbp*), primarily expressed by oligodendrocytes as a major component of the myelin sheath, serves as a marker of nerve myelination [23]. These two genes, *gfap* and *mbp*, expressed in other cell types within the nervous system, were also included.

Following exposure to PRO, all genes, except *mbp*, exhibited a common transcriptional response trend characterized by an initial increase followed by a slight reduction, albeit with varying relative fold-changes and statistical significance. Conversely, exposure to MXO resulted in the downregulation of all these genes. The transcriptional changes in genes related to the nervous system may contribute to alterations in swimming speed. However, when considering locomotor behavior, as well as the transcriptional responses of nervous system genes and the enzyme activities, the magnitude and direction of changes suggest that the mechanisms underlying neurobehavioral toxicity may differ between PRO and MXO, implying variations in the distribution and physiological functions of α- and β-adrenergic receptors. The limited understanding of the adrenergic system in fish impedes further elucidation of the neurotoxicity of these compounds. Additionally, it should be noted that several genes, including *nestin* and *shha*, are expressed in tissues beyond the nervous system [23]. Therefore, besides the antagonistic effects on adrenergic receptors, the observed neurobehavioral toxicity may also involve impacts on the overall developmental processes during the early life stages of zebrafish. Given that neurobehavioral toxicity is believed to result from various mechanisms of action, the potential of neurobehavioral effects to serve as validated biomarkers for exposure to drugs targeting adrenergic receptors warrants further investigation. Considering that sleep disorders and fatigue are recognized side effects of adrenergic receptor antagonists [7], future studies could explore their impacts on activity rhythms, sleep patterns, or even breeding cycles in fish, which hold greater ecological significance.

Although exposure to MOX and PRO in the early life stages of zebrafish showed only marginal effects on survival and development, significant impacts on cardiovascular and nervous system development were observed due to the specific actions of these pharmaceuticals. However, the persistence of these effects from early life stages into adulthood requires further investigation. Additionally, the simultaneous presence of a wide range of drugs targeting the adrenergic receptor in the aquatic environment, especially various β-blockers, warrants the paying of attention to potential additive or interactive effects of these mixtures. Previous studies suggest that β-blocker mixtures may exhibit higher toxicity than individual compounds, following either the concentration addition or independent action model [11]. Therefore, accurate ecotoxicological risk assessment for these pharmaceuticals remains an open question.

## 5. Conclusions

In summary, both α- and β-adrenergic receptor blockers induced detrimental effects on cardiovascular and nervous system development in the early life stages of zebrafish. The cardiovascular response could potentially serve as a biomarker of exposure to these blockers. However, the transcriptional responses of genes and enzyme activities differed between the two compounds, suggesting variations in distribution and physiological function of α- and β-adrenergic receptors in zebrafish. Comparative studies on the effects of α- and β-blockers in fish not only enhance our understanding of the ecotoxicological risks associated with these pharmaceuticals, but also provide insights into the adrenergic system and its physiological functions in fish.

## Figures and Tables

**Table 1:** Changes of vascular diameter, blood flow volume and heart rate in the zebrafish larvae following exposure to moxisylyte (MOX) and propranolol (PRO).

Treatment (μg/L)		Relative Vessel Diameter (%)	Relative Blood Flow Volume (%)	Relative Heart Rate (%)
MOX	0	100.0 ± 11.5	100.0 ± 4.0	100.0 ± 3.5
	1	94.6 ± 5.1	99.8 ± 3.9	108.5 ± 15.8
	5	98.8 ± 3.7	96.4 ± 4.7	111.7 ± 9.1
	25	90.5 ± 2.6	93.1 ± 5.1	97.9 ± 6.1
	125	87.3 ± 7.6	92.1 ± 7.7	93.2 ± 7.4
	625	80.3 ± 6.1 *	74.4 ± 11.8 **	85.4 ± 4.9 *
PRO	0	100.0 ± 5.7	100.0 ± 7.3	100.0 ± 4.3
	1	97.0 ± 4.3	101.1 ± 8.6	97.9 ± 6.1
	5	95.4 ± 4.7	101.4 ± 5.9	93.2 ± 3.4
	25	89.2 ± 5.6	88.7 ± 6.7	90.6 ± 4.8 *
	125	86.6 ± 3.3 *	80.2 ± 4.0 *	86.8 ± 2.0 **
	625	81.1 ± 5.2 **	76.5 ± 4.4 **	84.4 ± 3.5 **

The values relative to the controls are expressed as the mean ± S.D. (three replicates with fifteen larvae each). Asterisks denote significant differences compared to the controls: * *p* < 0.05 and ** *p* < 0.01.

## Data Availability

All data and materials are included in the manuscript.

## Supplementary Materials

The following supporting information can be downloaded at: https://www.mdpi.com/article/10.3390/toxics12080583/s1, Figure S1: Morphological malformation of zebrafish larvae following exposure to moxisylyte; Table S1: Developmental toxicity of moxisylyte (MOX) and propranolol (PRO) on zebrafish embryos.

## References

[1] Ciccarelli M., Sorriento D., Coscioni E., Iaccarino G., Santulli G., Schisler J.C., Lang C.H., Willis M.S. (2017). Chapter 11—Adrenergic Receptors. Endocrinology of the Heart in Health and Disease.

[2] Engelhardt S., Hein L., Offermanns S., Hein L. (2004). Adrenergic System. Transgenic Models in Pharmacology.

[3] Vardanyan R., Hruby V., Vardanyan R., Hruby V. (2016). Chapter 12—Adrenoblockers. Synthesis of Best-Seller Drugs.

[4] Gerald W., Dorn I. (2010). Adrenergic signaling polymorphisms and their impact on cardiovascular disease. Physiol. Rev..

[5] Massarsky A., Trudeau V.L., Moon T.W. (2011). β-blockers as endocrine disruptors: The potential effects of human β-blockers on aquatic organisms. J. Exp. Zool. A Ecol. Genet. Physiol..

[6] Maszkowska J., Stolte S., Kumirska J., Lukaszewicz P., Mioduszewska K., Puckowski A., Caban M., Wagil M., Stepnowski P., Bialk-Bielinska A. (2014). Beta-blockers in the environment: Part I. Mobility and hydrolysis study. Sci. Total. Environ..

[7] Owen S.F., Giltrow E., Huggett D.B., Hutchinson T.H., Saye J., Winter M.J., Sumpter J.P. (2007). Comparative physiology, pharmacology and toxicology of β-blockers: Mammals versus fish. Aquat. Toxicol..

[8] Patel M., Kumar R., Kishor K., Mlsna T., Pittman C.U., Mohan D. (2019). Pharmaceuticals of emerging concern in aquatic systems: Chemistry, occurrence, effects, and removal methods. Chem. Rev..

[9] Küster A., Adler N. (2014). Pharmaceuticals in the environment: Scientific evidence of risks and its regulation. Philos. Trans. R. Soc. Lond. B Biol. Sci..

[10] Kuster A., Alder A.C., Escher B.I., Duis K., Fenner K., Garric J., Hutchinson T.H., Lapen D.R., Pery A., Rombke J. (2010). Environmental risk assessment of human pharmaceuticals in the European Union: A case study with the beta-blocker atenolol. Integr. Environ. Assess. Manag..

[11] Yi M., Sheng Q., Sui Q., Lu H.J. (2020). Beta-blockers in the environment: Distribution, transformation, and ecotoxicity. Environ. Pollut..

[12] Santos L.H., Araújo A.N., Fachini A., Pena A., Delerue-Matos C., Montenegro M.C.B.S.M. (2010). Ecotoxicological aspects related to the presence of pharmaceuticals in the aquatic environment. J. Hazard. Mater..

[13] Sun L., Xin L., Peng Z., Jin R., Jin Y., Qian H., Fu Z. (2014). Toxicity and enantiospecific differences of two beta-blockers, propranolol and metoprolol, in the embryos and larvae of zebrafish (*Danio rerio*). Environ. Toxicol..

[14] Sun L., Liu F., Chen H., Wang S., Lin X., Chi J., Zhu Q., Fu Z. (2015). Transcriptional responses in adult zebrafish (*Danio rerio*) exposed to propranolol and metoprolol. Ecotoxicology.

[15] Wang H., Lin X., He Z., Qian B., Sun L. (2022). Effects of adrenergic alpha-antagonists on the early life stages of Japanese medaka (*Oryzias latipes*). Ecotoxicology.

[16] Sun L., Gu L., Tan H., Liu P., Gao G., Tian L., Chen H., Lu T., Qian H., Fu Z. (2019). Effects of 17α-ethinylestradiol on caudal fin regeneration in zebrafish larvae. Sci. Total. Environ..

[17] Westerfield M. (2000). The Zebrafish Book: A Guide for the Laboratory Use of Zebrafish (Danio rerio).

[18] OECD (2013). Test No. 236: Fish Embryo Acute Toxicity (FET) Test.

[19] Finn J., Hui M., Li V., Lorenzi V., de la Paz N., Cheng S.H., Lai-Chan L., Schlenk D. (2012). Effects of propranolol on heart rate and development in Japanese medaka (*Oryzias latipes*) and zebrafish (*Danio rerio*). Aquat. Toxicol..

[20] Marquer C., Bressolle F. (1998). Moxisylyte: A review of its pharma codynamic and pharmacokinetic properties, and its therapeutic use in impotence. Fundam. Clin. Pharmacol..

[21] Sun L., Xu W., Peng T., Chen H., Ren L., Tan H., Xiao D., Qian H., Fu Z. (2016). Developmental exposure of zebrafish larvae to organophosphate flame retardants causes neurotoxicity. Neurotoxicol. Teratol..

[22] Noyes P.D., Haggard D.E., Gonnerman G.D., Tanguay R.L. (2015). Advanced morphological—Behavioral test platform reveals neurodevelopmental defects in embryonic zebrafish exposed to comprehensive suite of halogenated and organophosphate flame retardants. Toxicol. Sci..

[23] Fan C.-Y., Cowden J., Simmons S.O., Padilla S., Ramabhadran R. (2010). Gene expression changes in developing zebrafish as potential markers for rapid developmental neurotoxicity screening. Neurotoxicol. Teratol..

[24] Wu Q., Yan W., Liu C., Li L., Yu L., Zhao S., Li G. (2016). Microcystin-LR exposure induces developmental neurotoxicity in zebrafish embryo. Environ. Pollut..

[25] Du Z., Wang G., Gao S., Wang Z. (2015). Aryl organophosphate flame retardants induced cardiotoxicity during zebrafish embryogenesis: By disturbing expression of the transcriptional regulators. Aquat. Toxicol..

[26] Livak K.J., Schmittgen T.D. (2001). Analysis of relative gene expression data using real-time quantitative PCR and the 2(T)(-Delta Delta C) method. Methods.

[27] Fraysse B., Mons R., Garric J. (2006). Development of a zebrafish 4-day toxicity of embryo-larval bioassay to assess chemicals. Ecotox. Environ. Saf..

[28] Larsson D.G.J., Fredriksson S., Sandblom E., Paxeus N., Axelsson M. (2006). Is heart rate in fish a sensitive indicator to evaluate acute effects of β-blockers in surface water?. Environ. Toxicol. Pharmacol..

[29] Dzialowski E., Turner P., Brooks B. (2006). Physiological and reproductive effects of beta adrenergic receptor antagonists in *Daphnia magna*. Arch. Environ. Contam. Toxicol..

[30] Stanley J.K., Ramirez A.J., Mottaleb M., Chambliss C.K., Brooks B.W. (2006). Enantiospecific toxicity of the beta-blocker propranolol to *Daphnia magna* and *Pimephales promelas*. Environ. Toxicol. Chem..

[31] Blader P., Lam C.S., Rastegar S., Scardigli R., Nicod J.C., Simplicio N., Plessy C., Fischer N., Schuurmans C., Guillemot F. (2004). Conserved and acquired features of neurogenin1 regulation. Development.

